# Determination of Fertility-Related Traits in Muscadine Grape Population

**DOI:** 10.3390/plants10061175

**Published:** 2021-06-09

**Authors:** Jiovan Campbell, Pranavkumar Gajjar, Ahmed Ismail, Fariborz Habibi, Ahmed G. Darwish, Violeta Tsolova, Ali Sarkhosh, Islam El-Sharkawy

**Affiliations:** 1 Center for Viticulture and Small Fruit Research, College of Agriculture and Food Sciences, Florida A&M University, Tallahassee, FL 32308, USA; jiovan1.campbell@famu.edu (J.C.); pranavkumar1.gajjar@famu.edu (P.G.); ahmed.ismail@agr.dmu.edu.eg (A.I.); ahmed.darwish@famu.edu (A.G.D.); violeta.tsolova@famu.edu (V.T.); 2 Department of Horticulture, Faculty of Agriculture, Damanhour University, Damanhour 22516, Egypt; 3 Department of Horticultural Science, School of Agriculture, Shiraz University, Shiraz 71441-65186, Iran; fariborz_h659@yahoo.com; 4 Department of Biochemistry, Faculty of Agriculture, Minia University, Minia 61519, Egypt; 5 Horticultural Sciences Department, University of Florida, Gainesville, FL 32611, USA; sarkhosha@ufl.edu

**Keywords:** fertility, flower, muscadine grape, yield

## Abstract

In this study, fertility-related traits of 90 muscadine grape genotypes were evaluated. Selected genotypes included 21 standard cultivars, 60 breeding lines, and nine *Vitis × Muscadinia* hybrids (VM hybrids). The first fruiting bud (FFB), bud fertility (BF), bud fertility coefficient (BFC), number of flowers/flower cluster (N.F/FC), fruit-set efficiency (FSE), number of clusters/vine (N.C/V), and yield/vine (Y/V) traits were evaluated. The FFB trait did not show significant differences among genotypes. The muscadine genotype O28-4-2-2 (1.6 ± 0.2) displayed the FFB closest to the base; however, O17-16-2-1, O18-2-1, and VM A12-10-2 genotypes had the most distant FFB (3.6 ± 0.3). All the other fertility-related traits varied widely among the population. The BF, BFC, N.F/FC, FSE, N.C/V, and Y/V exhibited a range estimated at 35.1%, 81.5%, 259.7, 63.3%, 177 C/V, and 22.3 kg/V, respectively. The muscadine genotypes O42-3-1 (36.7% ± 1.3) and Majesty (34% ± 1.2) exhibited the highest BF; however, the VM A12-10-2 (1.6% ± 0.1) recorded the lowest BF. The VM genotype O15-16-1 (82.8% ± 4.1) displayed the highest BFC; however, the VM A12-10-2 (1.3% ± 0.1) showed the lowest BFC. The muscadine genotypes D7-1-1 (280.3 F/FC ± 21.7) and O17-17-1 (20.7 F/FC ± 5.5) showed the highest and lowest N.F/FC, respectively. The maximum and minimum FSE was observed for the Rosa cultivar (65.7% ± 2.4) and muscadine genotype D7-1-1 (2.4% ± 0.2), respectively. The minimum N.C/V was recorded for VM genotype A12-10-2 (6 C/V ± 0.2) and maximum noted for muscadine genotypes B20-18-2 (183 C/V ± 7.5) and O44-14-1 (176 C/V ± 7.3). Muscadine genotype O23-11-2 (22.6 kg ± 1.1) produced the highest Y/V; however, the lowest yield was recorded for O15-17-1, Fry Seedless, Sugargate, and the VM genotypes and A12-10-2, with an average yield among them estimated at 0.4 kg ± 0.2.

## 1. Introduction

Stable productivity is an important goal of vineyards and depends on diverse components [1]. Achieving this outcome may become more critical in the near future due to the need to adapt viticulture to a changing climate [2,3,4]. The predominant *V. vinifera* industry faces severe pressures from pathogens and a rapidly changing environment. As a result, the intensity of agrochemical use has increased and the distribution of regions suitable for grapevine cultivation is becoming limited. Consequently, there is an urgent need to develop new cultivars that carry desirable adaptive traits capable of overcoming the existing and future challenges to the sustainability of the national grape industry. Muscadines (*Muscadinia rotundifolia* (Michx.) Small) are commonly grown grapes in the southeastern region of the United States due to their high adaptability to diverse biotic/abiotic stresses and unique fruit quality characteristics [5]. The recent recognition that muscadine grapes are major sources of health-promoting phytochemicals has considerably increased their demand by consumers as a desirable functional food [6]. Thus, the importance of the *Muscadinia* species is due to its commercial crop value and as source of germplasm for economically important traits.

Grapevine bud fertility is a primary component that influences the ultimate yield quantity and quality [7]. Bud fertility involves a complex and coordinated interplay of several critical components, which operate mutually to optimize the potential for forming fruiting buds. These components can be divided into environmental, vineyard practice management, and genetic factors.

Environmental factors, such as light intensity, temperature, and water availability, significantly influence bud fertility. High light intensity and temperatures promote the synthesis of gibberellin and cytokinin phytohormones, which stimulate the differentiation of buds into inflorescence primordia [8]. Gibberellin enhances Anlagen (uncommitted primordia) formation [9,10], whereas cytokinin induces the development of inflorescences from Anlagen [11]. In contrast, shading can provoke necrosis and bud death [12].

Excess shoot growth vigor can reduce bud fertility due to bud necrosis [13]. Therefore, the adoption of vineyard practice management to control vine vigor via pruning, which indirectly improves the exposure of buds to light, can increase bud fertility. Further, knowledge of the position of fertile buds in each genotype is essential for establishing a more rational pruning strategy that increases vineyard yield [14]. Additionally, control of irrigation is one of the most significant factors that can influence the level of bud fertility. For instance, excess water can result in more conflicting effects than water deficit because it promotes vegetative growth [12]. Other management factors, such as fertilization, can have a severe impact on bud fertility. The excessive use of nitrogen fertilization can change vine growth strategy via supporting vegetative growth rather than reproductive growth. Finally, bud fertility has a vital genetic component, which results in a wide variability of the trait among different genotypes [15]. Studies of the genetic elements of fertility variation are scarce, although they are necessary to improve this trait through breeding. The few available estimates of heritability for the number of clusters per shoot suggest that a relatively large portion of the phenotypic variation of this trait is of genetic origin, with greater dominance than additivity [7,15].

Overall, bud fertility is a critical trait that determines vineyard yield and must be carefully considered to develop and select new cultivars, and to ensure sustainable production. Therefore, the current study aimed to evaluate the diverse fertility-related traits of 90 muscadine grape genotypes over three production cycles. This study is the first research considering a population evaluation of fertility traits in muscadine grape. Further, we previously evaluated the characteristics of the same muscadine grape genotypes for other traits, including flower structure, cluster- and berry-related traits, biochemical, and color-related traits [16,17,18]. Accordingly, we provide a wide cross-correlation between the different evaluated traits.

## 2. Results and Discussion

### 2.1. First Fruiting Bud Trait

**Figure 1:**
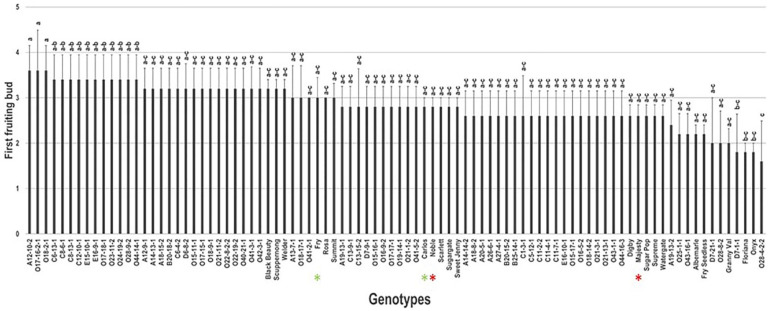
Characterization of first fruiting bud (FFB) trait among muscadine population (*n* = 90). The bars represent the mean FFB position (±SD) results from three biological replicates (three vines) over three years (*n* = 9). The y-axis refers to the position of the first fruiting bud. The x-axis refers to muscadine genotypes. Means within columns for the same letter followed by different letters differ significantly by Tukey’s test (*p* < 0.05). The asterisk refers to standard commercial cultivars selected as reference genotypes in the current study, including Noble, Carlos, Majesty, and Fry.

The first fruiting bud (FFB) trait was determined for a genotype based on identifying the first bud from the base that differentiates and develops into a flower cluster. Grapevines are pruned depending on the occurrence of flowering bud events on the new shoots. Spur-pruned grape varieties produce flower clusters on new growth from buds close to the base of one-year canes, near the main stem. However, cane-pruned grape varieties produce flower clusters on new growth from buds towards the end of one-year canes [19]. In general, all the characterized muscadine genotypes were able to produce commercial yield from basic buds. Accordingly, the FFB trait did not show significant differences among genotypes with a range of second bud position (1.6–3.6 buds) (Figure 1). On average, the FFB events occurred between the second and third bud (2.83 ± 0.4) with a median of FFB position at ~2.8. Statistical analysis designated that the muscadine genotype O28-4-2-2 (1.6 ± 0.3) displayed the closest FFB site to the base, with a fruiting bud event detected between the first and second bud. However, the muscadine genotypes O17-16-2-1, O18-2-1, and VM A12-10-2 had the most distant FFB from the bottom, with an FFB incident noticed between the 3rd and 4th bud (3.6 ± 0.6) from the base. For all commercial cultivars used as a reference for this study, including Carlos, Fry, Majesty, and Noble, the FFB event was observed between the second and third bud from the base.

### 2.2. Bud Fertility Trait

**Figure 2:**
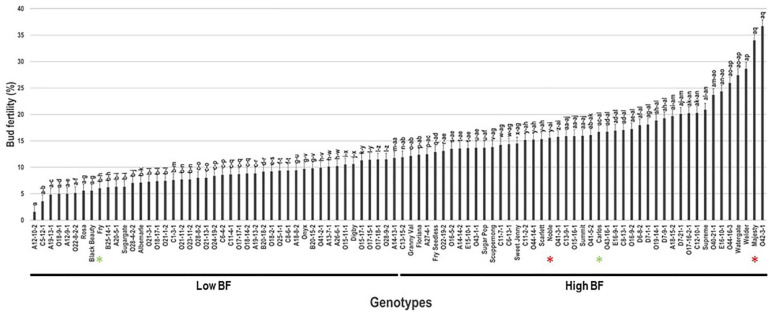
Characterization of bud fertility (BF) trait among muscadine population (*n* = 90). The bars represent the mean BF (±SD) results from three biological replicates (thee vines) over three years (*n* = 9). The y-axis refers to bud fertility (%). Means within columns for the same letter followed by different letters differ significantly by Tukey’s test (*p* < 0.05). Based on median BF (~11.8%), the population was divided into two groups that showed low and high BF. Other details are as in Figure 1.

Bud fertility (BF) trait widely varied between muscadine individuals. Among the population, the BF trait had an extensive range of 35.1% (Figure 2). The average BF for the population was estimated at 12.9% ± 6.5. Based on the median BF (~11.8%), the muscadine population was divided into two groups that showed low (44 genotypes, 48.9% of the population) and high bud fertility (46 genotypes, 51.1% of the population). Statistical analysis suggested that the VM genotype A12-10-2 (2.6% ± 0.2) had the lowest BF level. However, the muscadine genotype O42-3-1 (36.7% ± 1.3) exhibited the highest BF without any significant differences with the muscadine cultivar Majesty. According to the population analysis, the standard commercial cultivars, Majesty (34.0% ± 1.2), Carlos (16.7% ± 0.6), and Noble (15.6% ± 0.5), were classified as members of the muscadine group exhibiting high bud fertility level. However, Fry cultivar was a member of the low bud fertility group (6.0% ± 0.2).

### 2.3. Bud Fertility Coefficient Trait

**Figure 3:**
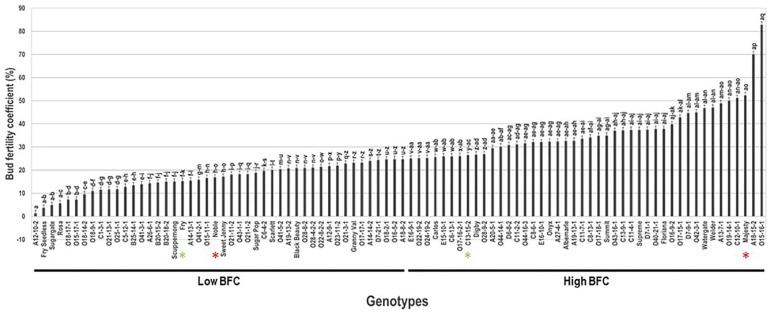
Characterization of bud fertility coefficient (BFC) trait among muscadine population (*n* = 90). The bars represent the mean BFC (±SD) results from three biological replicates (three vines) over three years (*n* = 9). The y-axis refers to the bud fertility coefficient (%). Means within columns for the same letter followed by different letters differ significantly by Tukey’s test (*p* < 0.05). Based on median BFC (~24.7%), the population was divided into two equal groups of high and low BFC. Other details are as in Figure 1.

Bud fertility coefficient (BFC) trait considerably differed within the population with a wide range of 81.5%. The average BFC level was estimated at 26.4% ± 13.8. The median BFC (~24.7%) divided the population into two equal groups of high and low bud fertility coefficient (Figure 3). The VM genotype A12-10-2 (1.3% ± 0.1) had the lowest BFC level. However, the VM hybrid genotype O15-16-1 displayed the highest BFC (82.8% ± 4.1). It is important to highlight that the cause of low BFC levels in several genotypes is due to the occurrence of flower cluster abortion. The BFC/BF ratio was less than 1.0 in seven VM and muscadine genotypes, including A12-10-2, O15-17-1, O18-17-1, O41-3-1, Fry Seedless, Rosa, and Sugargate. Population analysis placed the commercial cultivars Majesty (52.3% ± 2.6), Carlos (25.7% ± 1.3) among the genotypes displaying high bud fertility coefficient levels, whereas the Noble (16.8% ± 0.8) and Fry (15.3% ± 0.8) cultivars were members of the low bud fertility coefficient group.

### 2.4. Number of Flowers/Flower Cluster

**Figure 4:**
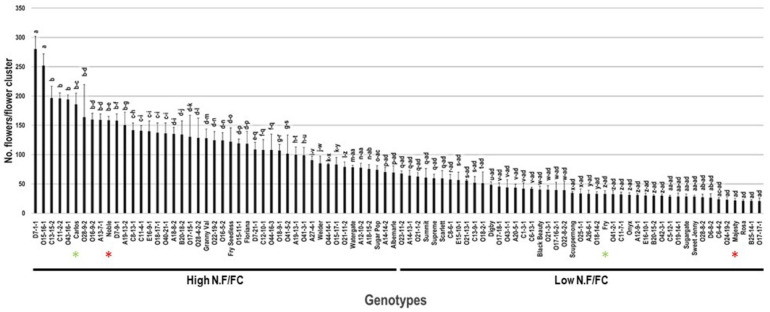
Characterization of the number of flowers/flower cluster (N.F/FC) trait among muscadine population (*n* = 90). The bars represent the mean N.F/FC (±SD) results from three biological replicates (three vines) over three years (*n* = 9). The y-axis refers to the number of flowers/flower cluster. Means within columns for the same letter followed by different letters differ significantly by Tukey’s test (*p* < 0.05). Based on median N.F/FC (~68.5 F/FC), the population was divided into two equal groups that displayed high and low clusters number. Other details are as in Figure 1.

The number of flowers/flower cluster (N.F/FC) trait had a broad range of 259.7 N.F/FC among the population. The muscadine D7-1-1 (280.3 F/FC ± 21.7) and the VM genotype O15-16-1 (252.0 F/FC ± 20.0) displayed the highest N.F/FC with insignificant differences between them. However, the five muscadine genotypes O24-19-2 (23.0 F/FC ± 5.0), Majesty (21.7 F/FC ± 4.0), Rosa (21.3 F/FC ± 2.1), B25-14-1 (21.0 F/FC ± 2.6), and O17-17-1 (20.7 F/FC ± 5.5) showed the lowest N.F/FC. The population had an average of 84.1 F/FC ± 56.5. Based on the median N.F/FC (~68.5 F/FC), the population was separated into two halves of high and low N.F/FC. Accordingly, the standard wine cultivars Carlos (186.0 F/FC ± 19.1) and Noble (158.7 F/FC ± 6.5) were classified among muscadine genotypes exhibiting high N.F/FC. However, Fry (32.7 F/FC ± 5.9) and Majesty table cultivars belonged to the group displaying low N.F/FC (Figure 4).

### 2.5. Fruit-Set Efficiency

**Figure 5:**
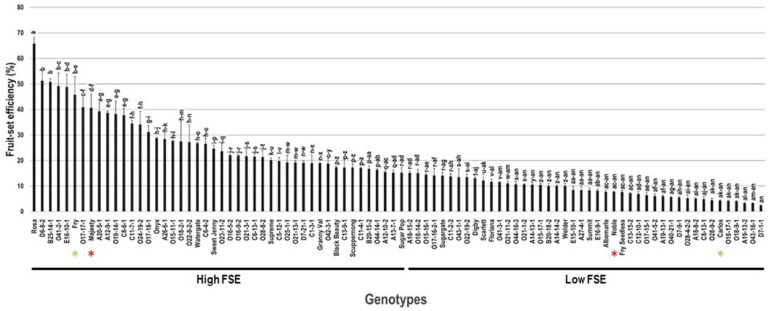
Characterization of the number of fruit-set efficiency (FSE) trait among muscadine population (*n* = 90). The bars represent the mean FSE (±SD) results from three biological replicates (three vines) over three years (*n* = 9). The y-axis refers to the fruit-set efficiency. Means within columns for the same letter followed by different letters differ significantly by Tukey’s test (*p* < 0.05). Based on median FSE (~15.2%), the population was divided into two groups of high (43 genotypes, or 47.8%) and low (47 genotypes, 52.2%) FSE. Other details are as in Figure 1.

The fruit-set efficiency (FSE) trait varied considerably among the population, with a wide range of 63.3%. The maximum FSE was recorded for the Rosa cultivar (65.7% ± 2.4), whereas the lowest FSE was observed in D7-1-1 (2.4% ± 0.2). The average FSE among the population was estimated at 18.7% ± 13.3. The median CWE (~15.2%) divided the population into two groups of high (43 genotypes, or 47.8%) and low (47 genotypes, 52.2%) FSE. The standard commercial table cultivars Fry (45.7% ± 7.1) and Majesty (40.6% ± 5.3) were classified as members of the muscadine group exhibiting high FSE. In contrast, the wine muscadines Noble (7.8% ± 0.2) and Carlos (4.3% ± 0.2) were members of the low FSE group (Figure 5).

### 2.6. Number of Clusters/Vine (N.C/V)

**Figure 6:**
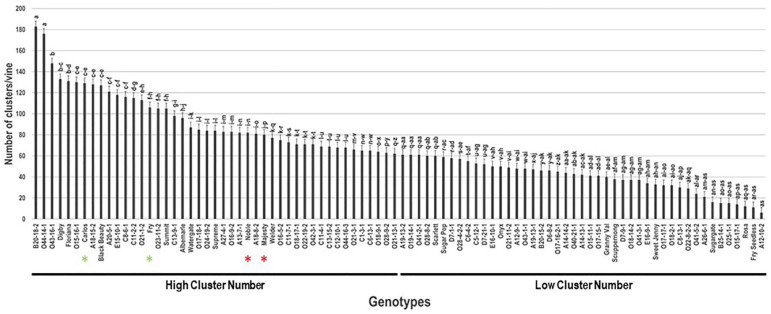
Characterization of number of clusters/vine (N.C/V) trait among muscadine population (*n* = 90). The bars represent the mean N.C/V (±SD) results from three biological replicates (three vines) over three years (*n* = 9). The y-axis refers to the number of clusters/vine. Means within columns for the same letter followed by different letters differ significantly by Tukey’s test (*p* < 0.05). Based on median N.C/V (~61.5 clusters), the population was divided into two equal groups that displayed high and low clusters number. Other details are as in Figure 1.

The average number of clusters/vine (N.C/V) was taken from three different vines/genotype. The N.C/V trait visibly varied among the population and displayed a wide range of 177 C/V (Figure 6). The average N.C/V was estimated at 67.7 C/V ± 36.9. The median N.C/V (~61.5 C/V) separated the population into two equal groups that displayed high and low clusters/vine. Muscadine genotypes B20-18-2 (183 C/V ± 5.5) and O44-14-1 (176 C/V ± 5.3) exhibited the highest N.C/V; however, the lowest N.C/V was recorded for the VM genotype A12-10-2 (6 C/V ± 0.2). Population analysis classified all the selected commercial cultivars, Carlos (129 C/V ± 11.6), Fry (106 C/V ± 9.5), Noble (82 C/V ± 7.4), and Majesty (80 C/V ± 7.2), among the genotypes displaying high clusters/vine. These results validated the selection of these genotypes as commercial cultivars. Accordingly, this trait can be used for the selection of new breeding lines to promote into advance selections and potential future cultivars upon confirmation of production stability and overall performance.

### 2.7. Yield/Vine (Y/V)

**Figure 7:**
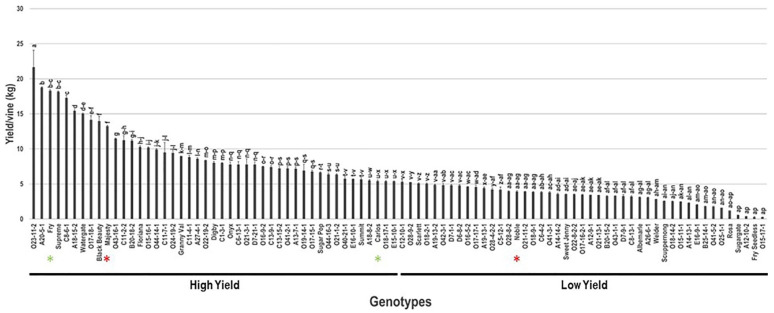
Characterization of yield/vine (Y/V) trait among muscadine population (*n* = 90). The bars represent the mean Y/V (±SD) results from three biological replicates (three vines) over three years (*n* = 9). The y-axis refers to the yield/vine (kg). Means within columns for the same letter followed by different letters differ significantly by Tukey’s test (*p* < 0.05). Based on median Y/V (~5.3 kg), the population was divided into two equal groups that showed high and low yield. Other details are as in Figure 1.

The average yield/vine (Y/V) was recorded upon reaching optimal harvest characteristics. Among the population, the Y/V trait had a wide range of 22.3 kg (Figure 7). Muscadine genotype O23-11-2 (22.6 kg ± 1.1) displayed the highest yield. However, the lowest Y/V was identified in several genotypes with no notable differences among them, including Sugargate (0.6 kg ± 0.2), Fry Seedless (0.3 kg ± 0.1), O15-17-1 (0.3 kg ± 0.1), and the VM genotype A12-10-2 (0.4 kg ± 0.1). The average Y/V among the population was estimated at 6.6 kg ± 4.5. Based on median Y/V (~5.3 kg/vine), the muscadine population was divided into two groups that exhibited high Y/V (46 genotypes, 51.1% of the population) and low Y/V (44 genotypes, 48.9% of the population). Population analysis positioned the commercial cultivars Fry (18.3 kg ± 1.6), Majesty (13.3 kg ± 1.2), and Carlos (5.4 kg ± 0.5) among genotypes displaying high Y/V, whereas the Noble (3.9 kg ± 0.4) cultivar was a member of the low Y/V group.

### 2.8. Distribution Frequency

**Figure 8:**
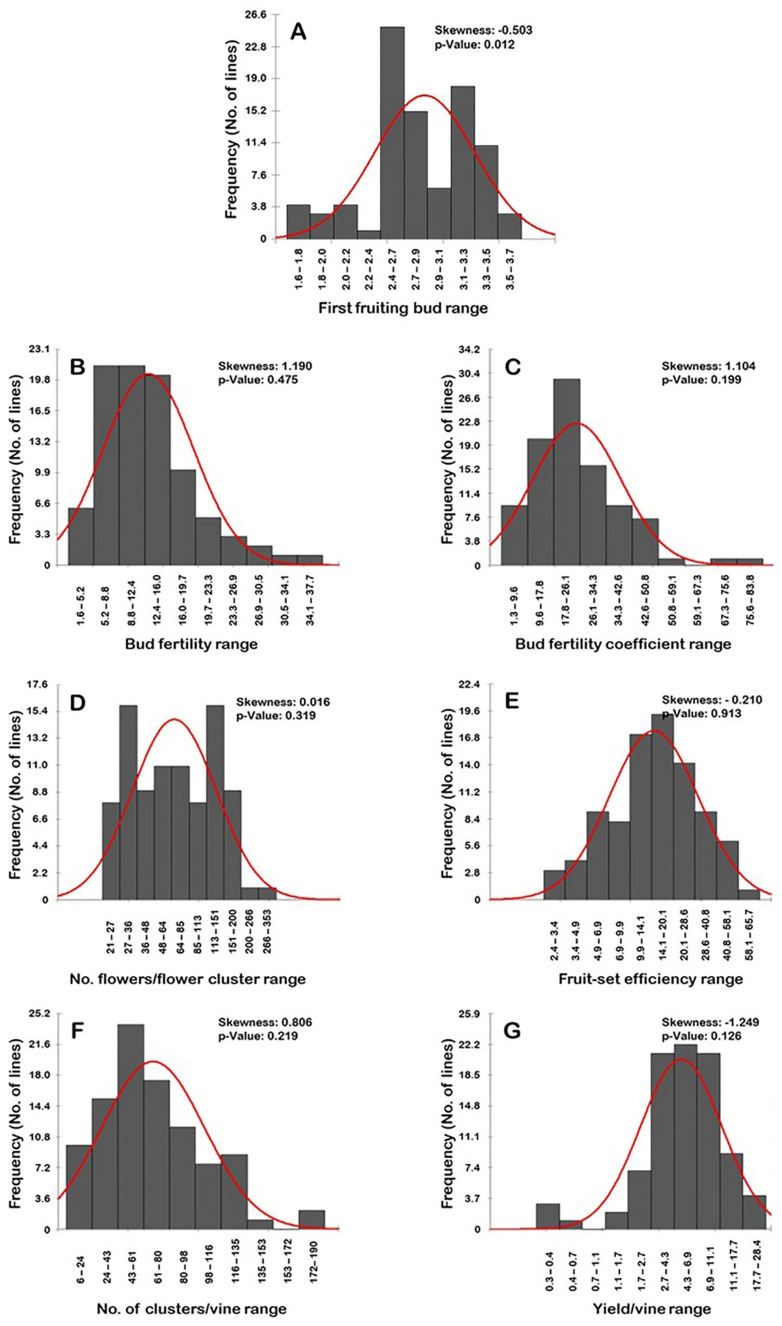
Frequency distribution of the fertility-related traits, first fruiting bud—FFB (**A**), bud fertility—BF (**B**), bud fertility coefficient—BFC (**C**), number of flowers/flower cluster—N.F/FC (**D**), fruit-set efficiency—FSE (**E**), number of clusters/vine—N.C/V (**F**), and yield/vine—Y/V (**G**) of the muscadine population (*n* = 90). The skewness degree and *p*-value of the Kolmogorov–Smirnov normal distribution test are indicated.

The frequency distribution of fertility-related traits was assessed (Figure 8). All the evaluated characteristics exhibited a normal frequency distribution pattern (*p* > 0.05), excluding the first fruiting bud (FFB) trait (Figure 8A). The distribution behavior for the bud fertility (BF), bud fertility coefficient (BFC), number of flowers/flower cluster (N.F/FC), and number of clusters/vine (N.C/V) were skewed to the right, departing from normality (Figure 8B–D,F). By contrast, the distribution pattern for the FFB, fruit-set efficiency (FSE), and yield/vine (Y/V) characteristics were skewed to the left, departing from normality (Figure 8A,E,G). However, the level of skewness visibly varied among traits, stretching from strongly (i.e., BF, BFC, N.C/V, and Y/V) to moderately (i.e., FFB) and slightly skewed (i.e., N.F/FC and FSE).

The BF trait followed a unimodal frequency distribution pattern by which two major BF modes were detected. The two modes were described as low BF (5.2–8.8%) and slightly higher BF (>8.8–12.4%). Each of these two modes was represented by 21 genotypes or ~23.3% of the population (Figure 8B).

The distribution pattern of BFC, FSE, and Y/V traits followed a bimodal frequency distribution (Figure 8C,E,G), suggesting two main phenotypes in the population. The two detected phenotypes for the BFC trait were designated as low to high BFC percentage (1.3–59.1%; 88 genotypes; ~97.8% of the population) and extremely high BFC levels (67.3–83.8%; two genotypes; ~2.2% of the population) (Figure 8C). The BFC range of 17.8–26.1% described the major mode (29 genotypes or ~32.2% of the population); however, the minor mode was observed within the range of 67.3–75.6% (one genotype or 1.1% of the population). The two phenotypes for the FSE trait were designated as low FSE percentage (2.4–6.9%; 16 genotypes; ~17.8% of the population) and modest to high FSE levels (> 6.9–65.7%; 74 genotypes; ~82.2% of the population) (Figure 8E). The major FSE mode among the population was observed for the intermediate FSE levels with a range of 14.1–20.1% (19 genotypes or ~21.1% of the population); however, the minor mode was detected for the low FSE levels with a range of 4.9–6.9% (nine genotypes or ~10.0% of the population). The two phenotypes for Y/V were designated as extremely low Y/V (0.3–0.7 kg; four genotypes; ~4.4% of the population) and low to high Y/V (1.7–28.4 kg; 86 genotypes; ~95.6% of the population) (Figure 8G). The major Y/V mode among the population was observed for the modest Y/V with a range of 4.3–6.9 kg (22 genotypes or ~24.4% of the population); however, the minor mode was detected for the low Y/V with a range of 0.3–0.4 kg (three genotypes or ~3.3% of the population).

The traits of N.F/FC and N.C/V typically have a trimodal distribution. A trimodal distribution usually indicates three main phenotypes in the studied population (Figure 8D,F). In the N.F/FC trait (Figure 8D), the three categories were designated as low flower number (21–36 F/FC; 24 genotypes or ~26.7% of the population), intermediate flower number (>36–113 F/FC; 39 genotypes or ~43.3% of the population), and a high flower number (>113–353 F/FC; 27 genotypes or ~30.0% of the population). The N.F/FC exhibited a particular mode among the population due to the detection of two dominant major modes. The two modes were observed for the low N.F/FC with a range of 27–36 F/FC and the high N.F/FC with a range of 113–151 F/FC. Each of these two major modes was represented by 16 genotypes or ~17.8% of the population. Similarly, two minor modes were detected for the intermediate number of flowers with the ranges of 48–64 F/FC and >64–85 F/FC. Each of these two minor modes was represented by 11 genotypes or ~12.2% of the population. In the N.C/V trait (Figure 8F), the three categories were designated as low to modest N.C/V (6–116 C/V; 79 genotypes or ~87.8% of the population), high N.C/V (>116–153 C/V; nine genotypes or ~10.0% of the population), and extremely high N.C/V (172–190 C/V; two genotypes or ~2.2% of the population). The major N.C/V mode was detected for the modest N.C/V with a range of 43–61 C/V (22 genotypes or ~24.4% of the population). The two minor modes were observed for the high N.C/V with a range of 116–135 C/V (eight genotypes or ~8.9% of the population) and the extremely high N.C/V with a range of 172–190 C/V (two genotypes or ~2.2% of the population).

Finally, the FFB trait exhibited a multimodal distribution pattern with four phenotypic categories, exhibiting FFB ranges of 1.6–2.0 (seven genotypes or ~7.8% of the population), >2.0–2.4 (five genotypes or ~5.6% of the population), >2.4–3.1 (46 genotypes or ~51.1% of the population), and >3.1–3.7 (32 genotypes or ~35.5% of the population) (Figure 8A). The major FFB mode described genotypes with an FFB range of 2.4–2.7 (25 genotypes or ~27.8% of the population). The three minor modes were observed for FFB ranges of 1.6–1.8 (four genotypes or ~4.4% of the population), 2.0–2.2 (four genotypes or ~4.4% of the population), and 3.1–3.3 (18 genotypes or ~20.0% of the population).

### 2.9. Cross-Correlation Analysis between Muscadine Traits

**Figure 9:**
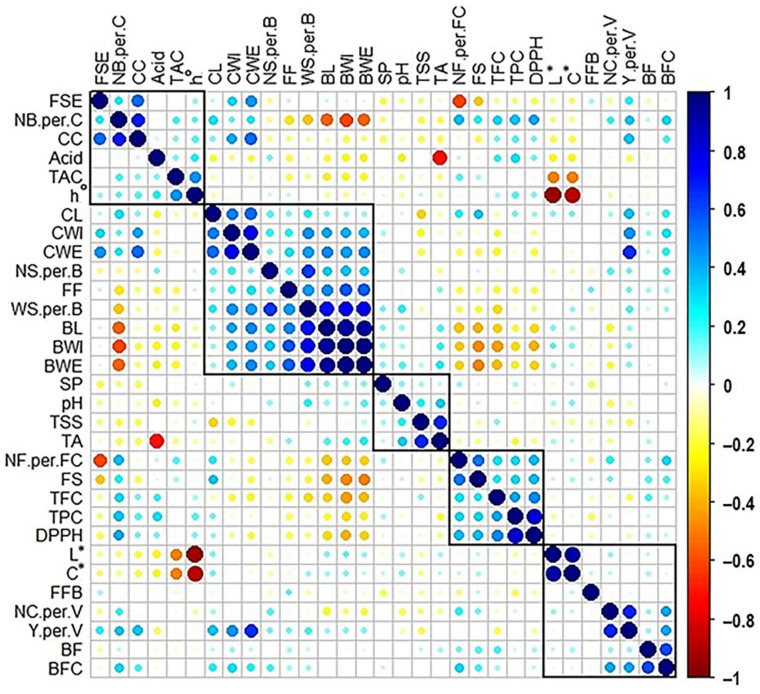
Pairwise Pearson correlation matrix of the fertility-related traits along with physical cluster- and berry-related traits [16], biochemical-, color-, and nutraceutical-related traits [17,18] in muscadine population (*n* = 90). By using hierarchical clustering, rectangles were drawn around traits data. Data are presented as an average from three biological replicates for three consecutive years. For the fertility-related traits; first fruiting bud (FFB), bud fertility (BF), bud fertility coefficient (BFC), number of flowers per flower cluster (NF.per.FC), fruit-set efficiency (FSE), number of clusters per vine (NC.per.V), and yield per vine (Y.per.V). The previously characterized traits include flower structure (FS), cluster length (CL), cluster width (CWI), cluster weight (CWE), number of berries per cluster (NB.per.C), cluster compactness (CC), berry length (BL), berry width (BWI), berry weight (BWE), number of seeds per berry (NS.per.B), weight of seeds per berry (WS.per.B), firmness (FF), scar pattern (SP), total soluble solids (TSS), titratable acidity (Acid), TSS/acid ratio (TA), pH, total anthocyanin content (TAC), luminosity index (L*), hue angle (h°), chroma index (C*), total phenolic content (TPC), total flavonoid content (TFC), and the 2,2-diphenyl-1-picryl-hydrazyl-hydrate (DPPH). The color change is proportional to the correlation level (see the color scale). The positive correlation is displayed in cyan and blue, the negative correlation in yellow and red.

To assess the correlation between the fertility-related traits and the previously evaluated characteristics in the 90 muscadine grape genotypes [16,17,18], Pearson’s correlation coefficients were determined (Figure 9). These cover a total of 31 traits, including flower structure (FS), cluster-related traits (cluster length (CL), width (CWI), weight (CWE), number of berries/cluster (NB.per.C), and compactness (CC)], berry-related traits [berry length (BL), berry width (BWI), berry weight (BWE), number of seeds/berry (NS.per.B), weight of seeds/berry (WS.per.B), firmness (FF), and scar pattern (SP)), biochemical-related traits (total soluble solids (TSS), titratable acidity (Acid), TSS/Acid ratio (TA), and pH), color-related traits (total anthocyanin content (TAC), luminosity index (L*), hue angle (h°), and chroma index (C*)), and nutraceutical-related traits (total phenolic content (TPC), total flavonoid content (TFC), and the 2,2-diphenyl-1-picryl-hydrazyl-hydrate (DPPH)).

The correlation plot was separated into five groups based on their significant correlation. The fruit-set efficiency (FSE) was a member of the first group, which also includes NB.per.C, CC, Acid, TAC, and h°. The FSE trait exhibited positive correlation with the traits of yield/vine (Y.per.V) (*r* = 0.224; P = 3.4 × 10^−2^), CWI (*r* = 0.314; P = 2.6 × 10^−3^), CWE (*r* = 0.448; P = 9.6 × 10^−6^), NB.per.C (*r* = 0.261; P = 1.3 × 10^−2^), and CC (*r* = 0.530; P = 7.3 × 10^−8^). However, the FSE trait was negatively correlated with the number of flowers/flower cluster (NF.per.FC) (*r* = −0.603; P = 3.1 × 10^−10^) and the perfect FS (*r* = −0.348; P = 7.7 × 10^−4^). The previous results suggested that the high fruit-set efficiency is associated with female flower structure and low number of flowers/flower cluster, which obviously influence the number of berries/cluster, cluster size, weight, compactness, and ultimate yield.

The NF.per.FC trait was placed among the fourth group along with FS, TFC, TPC, and DPPH traits. The NF.per.FC showed positive correlation with the bud fertility coefficient (BFC) (*r* = 0.316; P = 2.4 × 10^−3^), NC.per.V (*r* = 0.231; P = 2.9 × 10^−2^), perfect FS (*r* = 0.501; P = 4.9 × 10^−7^), CL (*r* = 0.253; P = 1.6 × 10^−2^), NB.per.C (*r* = 0.367; P = 3.8 × 10^−4^), TPC (*r* = 0.316; P = 2.4 × 10^−3^), TFC (*r* = 0.282; P = 7.0 × 10^−3^), and DPPH (*r* = 0.373; P = 2.9 × 10^−4^). However, NF.per.FC trait was negatively correlated with BL (*r* = −0.358; P = 5.4 × 10^−4^), BWI (*r* = −0.342; P = 9.8 × 10^−4^), and BWE (*r* = −0.332; P = 1.4 × 10^−3^). These results indicate that the high number of flowers/flower cluster is associated with perfect flower structure, which enhances bud fertility coefficient, number of clusters/vine, and number of berries/cluster. However, it was associated with reduced berry characteristics in terms of size and weight. In fact, the previous enhanced traits define the general characteristics of muscadine grapes suitable for wine production. Interestingly, these characteristics were found to be associated with accelerated nutraceutical qualities in terms of phenolic/flavonoid content and the antioxidant activity.

All the remaining fertility traits, including first fruiting bud (FFB), bud fertility (BF), bud fertility coefficient (BFC), number of clusters/vine (NC.per.V), and yield/vine (Y.per.V) were grouped together with the color-related traits of L* and C*. The FFB trait did not display a major correlation with any of the evaluated traits. The BF trait showed a positive correlation with only BFC character (*r* = 0.584; P = 1.5 × 10^−9^). However, the BFC exhibited a similar positive correlation with both the NC.per.V and Y.per.V (*r* = 0.418; P = 4.1 × 10^−5^), NB.per.C (*r* = 0.305; P = 3.4 × 10^−3^), CL (*r* = 0.219; P = 3.8 × 10^−2^), CWI (*r* = 0.299; P = 4.1 × 10^−3^), and CWE (*r* = 0.227; P = 3.2 × 10^−2^). The NC.per.V trait showed a positive correlation with the Y.per.V trait (*r* = 0.672; P = 4.2 × 10^−13^) and a negative correlation with the BL (*r* = −0.222; P = 3.6 × 10^−2^). Finally, the Y.per.V trait showed a positive correlation with CL (*r* = 0.363; P = 4.5 × 10^−4^), CWI (*r* = 0.456; P = 6.3 × 10^−6^), CWE (*r* = 0.648; P = 5.0 × 10^−12^), NB.per.C (*r* = 0.339; P = 1.1 × 10^−3^), CC (*r* = 0.353; P = 6.4 × 10^−4^), and FF (*r* = 0.239; P = 2.3 × 10^−2^). The previous results suggested a positive cross-correlation between the bud fertility coefficient, the number of clusters/vine, and the yield. However, the increased number of clusters/vine was associated with reduced berry length. The ultimate yield/vine character was the result of accelerated cluster characteristics in terms of size, weight, number of berries/cluster, and berry firmness. However, these characteristics obviously produce compact clusters.

## 3. Materials and Methods

### 3.1. Plant Material

The experiment was carried out in the Center for Viticulture, Tallahassee, Florida (30°28′45.63″ N, 84°10′16.43″ W) over three production cycles in the period between 2017 and 2019.

Pruning was carried out each winter between January and March. It was performed by leaving the spurs with three to five buds near the primary arm of the vine. Other vineyard management and practices followed the guidelines outlined in the Muscadine Production Guide for Florida written by Florida Agricultural & Mechanical University (FAMU), Center for Viticulture and Small Fruit Research (CVSFR) (http.//famu.edu/viticulture, accessed on 20 January 2018).

The muscadine population used in this study was generated as part of the grape breeding program at the CVSFR, FAMU. For this study, 90 muscadine genotypes, including 21 standard cultivars, 60 breeding lines, and 9 *Vitis × Muscadinia* hybrids (VM hybrids), were selected based on vines’ age with at least 5 year old vines to ensure stable productivity. A complete list of used genotypes is presented (Table S1).

### 3.2. Fertility-Related Traits

During winter and after pruning time, the total number of buds per vine for each genotype was recorded (*n* = 3 vines). During the phenological phase of visible inflorescences, in shoots with approximately 5 leaves and 10 cm length, according to Coombs [20], the position of fruiting shoots on the bearing units of each vine was recorded to describe the first fruiting bud (FFB) trait of each genotype. The total number of fruiting buds, defined as the buds that produce flower clusters, were counted. Counting was performed for each bud position from the basal bud to the terminal bud of the cane. The total number of flowers/flower cluster (N.F/FC) was counted for each genotype at closed flower stage. The fruit-set efficiency (FSE) trait was calculated, as follows:

FSE (%) = (No. of berries/No. of flowers) × 100(1)

where the number of berries represents the berries number/cluster counted at post-veraison stage.

Finally, the total number of clusters per vine was counted at the veraison stage, when the cluster is well established and passing the period of cluster abortion. Bud fertility (BF) was obtained by calculating the ratio between the number of fruiting buds/vine and the total number of buds/vine. However, the bud fertility coefficient (BFC) trait was obtained by calculating the ratio between the number of clusters/vine and the total number of buds/vine. Due to unfavorable weather circumstances of heavy rain during the growing season, which can cause cluster/berry loss, we generated an equation to calculate the yield/vine accurately, as follows:

Yield/vine (kg) = [Average weight of cluster (g) × No. of clusters/vine]/1000(2)

### 3.3. Statistical Analysis

Data of several evaluated traits were collected and analyzed to test the genotype effect using repeated measures of analysis of variance (ANOVA) in SAS (SAS version 9.4, SAS Institute Inc., Raleigh, NC, USA) using PROC GLIMMIX. Means for the analyses were determined using the LSMEANS statement and means separation conducted using the Tukey–Kramer adjusted multiple means comparison test. All data are presented as the mean ± SD of 3 biological replicates. The R package corrplot was used to produce the correlation matrix plot [21].

## 4. Conclusions

Overall, the results of our study highlight that the evaluated muscadine genotypes are suitable for use in a genome-wide association study to investigate the genetic basis controlling most of the muscadine quality traits assessed in the current and previous studies [16,17,18]. The diversity in BF, BFC, N.C/V, and Y/V suggests a potent improvement in these traits via breeding. The fertility-related traits of 12 muscadine genotypes identified during this study are shown in Table 1. In addition to the four control standard cultivars, this includes seven muscadine-breeding lines, and one VM hybrid. Only the standard cultivar Noble exhibited low yield/vine based on the population average. This might occur due to low BFC levels, which are associated with the production of small berries and clusters. Compared with the standard cultivars, Fry and Majesty, the breeding lines A18-15-2 (bronze), O23-11-2 (dark red), and O43-16-1 (bronze) are considered promising new cultivars for fresh consumption. Nonetheless, none of these cultivars’ berry size reached that of the Majesty cultivar. Relative to the standard cultivars, Carlos and Noble, the muscadine genotypes A20-5-1 (bronze), B20-18-2 (black), C8-6-1 (bronze), and O44-14-1 (bronze), and the VM O15-16-1 (red), are considered promising new cultivars for wine production.

## Figures and Tables

**Table 1:** Characteristics of selected genotypes to promote new standard cultivars.

Genotype	Bud Fertility (%)	Bud Fertility Coefficient (%)	No. Flowers/Flower Cluster	Fruit-Set Efficiency	No. Clusters/Vine	Yield/Vine (kg)	Berry Weight (g)	Cluster Weight (g)
Carlos	16.7 ± 0.6	25.6 ± 1.3	186.0 ± 19.1	4.3 ± 0.2	129 ± 3.9	5.4 ± 0.3	6.7 ± 0.5	41.9 ± 2.9
Noble	15.6 ± 0.5	16.8 ± 0.8	158.7 ± 6.5	7.8 ± 0.2	82 ± 2.5	3.9 ± 0.2	4.2 ± 0.3	47.8 ± 3.3
Fry	6.0 ± 0.2	15.3 ± 0.8	32.7 ± 5.9	45.7 ± 7.1	106 ± 3.2	18.3 ± 0.9	12.7 ± 0.9	172.8 ± 12.1
Majesty	34.0 ± 1.2	52.3 ± 2.6	21.7 ± 4.0	40.6 ± 5.3	80 ± 2.4	13.3 ± 0.7	27.6 ± 1.9	165.7 ± 11.6
A18-15-2	19.7 ± 0.7	69.9 ± 3.5	75.3 ± 7.8	15.1 ± 0.8	128 ± 3.8	15.4 ± 0.8	13.3 ± 0.9	120.5 ± 8.4
A20-5-1	6.3 ± 0.2	29.4 ± 1.5	43.7 ± 5.9	39.2 ± 3.4	121 ± 3.6	18.8 ± 0.9	9.4 ± 0.7	155.2 ± 10.9
B20-18-2	9.2 ± 0.3	15.0 ± 0.8	134.3 ± 23.2	10.0 ± 1.0	183 ± 5.5	11.2 ± 0.6	5.2 ± 0.4	61.1 ± 4.3
C8-6-1	9.4 ± 0.3	32.0 ± 1.6	57.7 ± 8.5	37.7 ± 2.9	116 ± 3.5	17.3 ± 0.9	8.6 ± 0.6	149.1 ± 10.4
O15-16-1	15.9 ± 0.6	82.8 ± 4.1	252.0 ± 20.0	14.4 ± 0.6	130 ± 3.9	10.2 ± 0.5	3.3 ± 0.2	78.3 ± 5.5
O23-11-2	7.7 ± 0.3	21.9 ± 1.1	67.7 ± 4.7	23.6 ± 2.9	105 ± 3.2	22.5 ± 1.1	14.1 ± 1.0	214.8 ± 15.0
O43-16-1	16.7 ± 0.6	36.9 ± 1.8	194 ± 8.2	3.2 ± 0.2	148 ± 4.4	11.5 ± 0.6	15.4 ± 1.2	77.5 ± 5.4
O44-14-1	15.2 ± 0.5	30.1 ± 1.5	83.7 ± 1.5	16.4 ± 0.4	176 ± 5.3	9.9 ± 0.5	4.6 ± 0.3	56.4 ± 3.9
Average *	12.9 ± 6.5	26.4 ± 13.8	84.1 ± 56.5	18.7 ± 13.3	67.7 ± 36.9	6.6 ± 4.5	12.2 ± 5.6	96.9 ± 42.7

* The average is the mean among the population resulting from three biological replicates (vines) over three years of the experiment.

## Data Availability

The data presented in this study are available to anyone up to request.

## Supplementary Materials

The following are available online at https://www.mdpi.com/article/10.3390/plants10061175/s1, Table S1. Muscadine genotypes used, and corresponding genetic background and type of flower.
