# Supplementary material for: Determination of Fertility-Related Traits in Muscadine Grape Population
Source: Plants (Basel). 2021 Jun 9;10(6):1175. doi: 10.3390/plants10061175 (PMC8227160; doi:10.3390/plants10061175)
Supplement: Supplementary file 1 [file plants-10-01175-s001.zip › plants-1236364-supplementary.pdf]

Sample #	Genotype	Species	Flower Structure
1	A12-9-1	Muscadine hybrid	Hermaphroditic (Perfect)
2	A12-10-2	Muscadine hybrid	Hermaphroditic (Perfect)
3	A13-7-1	Muscadine hybrid	Hermaphroditic (Perfect)
4	A14-13-1	Muscadine rotundifolia	Hermaphroditic (Perfect)
5	A14-14-2	Muscadine rotundifolia	Hermaphroditic (Perfect)
6	A18-8-2	Muscadine rotundifolia	Hermaphroditic (Perfect)
7	A18-15-2	Muscadine rotundifolia	Hermaphroditic (Perfect)
8	A19-13-1	Muscadine rotundifolia	Female
9	A19-13-2	Muscadine rotundifolia	Hermaphroditic (Perfect)
10	A20-5-1	Muscadine rotundifolia	Hermaphroditic (Perfect)
11	A26-6-1	Muscadine rotundifolia	Hermaphroditic (Perfect)
12	A27-4-1	Muscadine rotundifolia	Hermaphroditic (Perfect)
13	B20-15-2	Muscadine rotundifolia	Female
14	B20-18-2	Muscadine rotundifolia	Hermaphroditic (Perfect)
15	B25-14-1	Muscadine rotundifolia	Female
16	C1-3-1	Muscadine rotundifolia	Female
17	C5-12-1	Muscadine rotundifolia	Female
18	C6-4-2	Muscadine rotundifolia	Female
19	C6-13-1	Muscadine rotundifolia	Hermaphroditic (Perfect)
20	D6-8-2	Muscadine rotundifolia	Hermaphroditic (Perfect)
21	C8-6-1	Muscadine rotundifolia	Female
22	C8-13-1	Muscadine rotundifolia	Hermaphroditic (Perfect)
23	C11-2-2	Muscadine rotundifolia	Hermaphroditic (Perfect)
24	C11-4-1	Muscadine rotundifolia	Hermaphroditic (Perfect)
25	C11-7-1	Muscadine rotundifolia	Female
26	C12-10-1	Muscadine rotundifolia	Hermaphroditic (Perfect)
27	C13-9-1	Muscadine rotundifolia	Hermaphroditic (Perfect)
28	C13-15-2	Muscadine rotundifolia	Hermaphroditic (Perfect)
29	D7-1-1	Muscadine rotundifolia	Hermaphroditic (Perfect)
30	D7-9-1	Muscadine rotundifolia	Hermaphroditic (Perfect)
31	D7-21-1	Muscadine rotundifolia	Hermaphroditic (Perfect)
32	E15-10-1	Muscadine rotundifolia	Female
33	E16-9-1	Muscadine rotundifolia	Hermaphroditic (Perfect)
34	E16-10-1	Muscadine rotundifolia	Female
35	O15-11-1	Muscadine hybrid	Hermaphroditic (Perfect)
36	O15-16-1	Muscadine hybrid	Hermaphroditic (Perfect)
37	O15-17-1	Muscadine rotundifolia	Hermaphroditic (Perfect)
38	O16-5-2	Muscadine hybrid	Hermaphroditic (Perfect)
39	O16-9-2	Muscadine hybrid	Hermaphroditic (Perfect)
40	O17-15-1	Muscadine rotundifolia	Female

41	O17-16-2-1	Muscadine rotundifolia	Hermaphroditic (Perfect)
42	O17-17-1	Muscadine rotundifolia	Female
43	O17-18-1	Muscadine rotundifolia	Hermaphroditic (Perfect)
44	O18-2-1	Muscadine rotundifolia	Hermaphroditic (Perfect)
45	O18-9-1	Muscadine rotundifolia	Hermaphroditic (Perfect)
46	O18-14-2	Muscadine rotundifolia	Female
47	O18-17-1	Muscadine rotundifolia	Hermaphroditic (Perfect)
48	O19-14-1	Muscadine rotundifolia	Hermaphroditic (Perfect)
49	O21-1-2	Muscadine rotundifolia	Hermaphroditic (Perfect)
50	O21-3-1	Muscadine rotundifolia	Hermaphroditic (Perfect)
51	O21-11-2	Muscadine hybrid	Hermaphroditic (Perfect)
52	O21-13-1	Muscadine hybrid	Hermaphroditic (Perfect)
53	O22-8-2-2	Muscadine rotundifolia	Hermaphroditic (Perfect)
54	O22-19-2	Muscadine rotundifolia	Hermaphroditic (Perfect)
55	O23-11-2	Muscadine rotundifolia	Hermaphroditic (Perfect)
56	O24-19-2	Muscadine rotundifolia	Female
57	O25-1-1	Muscadine rotundifolia	Female
58	O28-4-2-2	Muscadine rotundifolia	Hermaphroditic (Perfect)
59	O28-8-2	Muscadine rotundifolia	Female
60	O28-9-2	Muscadine rotundifolia	Hermaphroditic (Perfect)
61	O40-21-1	Muscadine rotundifolia	Hermaphroditic (Perfect)
62	O41-2-1	Muscadine rotundifolia	Hermaphroditic (Perfect)
63	O41-3-1	Muscadine rotundifolia	Hermaphroditic (Perfect)
64	O41-5-2	Muscadine rotundifolia	Hermaphroditic (Perfect)
65	O42-3-1	Muscadine rotundifolia	Hermaphroditic (Perfect)
66	O43-1-1	Muscadine rotundifolia	Hermaphroditic (Perfect)
67	O43-16-1	Muscadine rotundifolia	Hermaphroditic (Perfect)
68	O44-14-1	Muscadine rotundifolia	Hermaphroditic (Perfect)
69	O44-16-3	Muscadine rotundifolia	Hermaphroditic (Perfect)
70	Albemarle	Muscadine rotundifolia	Hermaphroditic (Perfect)
71	Black Beauty	Muscadine rotundifolia	Female
72	Carlos	Muscadine rotundifolia	Hermaphroditic (Perfect)
73	Digby	Muscadine rotundifolia	Hermaphroditic (Perfect)
74	Floriana	Muscadine rotundifolia	Hermaphroditic (Perfect)
75	Fry	Muscadine rotundifolia	Female
76	Fry Seedless	Muscadine rotundifolia	Hermaphroditic (Perfect)
77	Granny Val	Muscadine rotundifolia	Hermaphroditic (Perfect)
78	Majesty	Muscadine rotundifolia	Female
79	Noble	Muscadine rotundifolia	Hermaphroditic (Perfect)
80	Onyx	Muscadine rotundifolia	Female
81	Rosa	Muscadine rotundifolia	Female

82	Scarlett	Muscadine rotundifolia	Female
83	Scuppernong	Muscadine rotundifolia	Female
84	Sugargate	Muscadine rotundifolia	Female
85	Sugar Pop	Muscadine rotundifolia	Female
86	Summit	Muscadine rotundifolia	Female
87	Supreme	Muscadine rotundifolia	Female
88	Sweet Jenny	Muscadine rotundifolia	Female
89	Watergate	Muscadine rotundifolia	Hermaphroditic (Perfect)
90	Welder	Muscadine rotundifolia	Hermaphroditic (Perfect)
